# Supplementary material for: Developing a Diagnostic Decision Support System for Benign Paroxysmal Positional Vertigo Using a Deep-Learning Model
Source: J Clin Med. 2019 May 8;8(5):633. doi: 10.3390/jcm8050633 (PMC6571642; doi:10.3390/jcm8050633)
Supplement: Supplementary file 1 [file jcm-08-00633-s001.zip › S data.pdf]

Developing a diagnostic decision support system for benign paroxysmal positional vertigo using a deep-learning model

Eun-Cheon Lim[1,2], Jeong Hye Park[1,2], Han Jae Jeon[3], Hyung-Jong Kim[1], Hyo-Jeong Lee[1,2], Chang-Geun Song[3], and Sung Kwang Hong[1,2] *

[1]Department of Otorhinolaryngology-Head and Neck Surgery, Hallym University College of Medicine, Anyang, Republic of Korea

[2]Laboratory of Brain & Cognitive Sciences for Convergence Medicine, Hallym University College of Medicine, Anyang, Republic of Korea

[3]Department of Convergence Software, Hallym University, Chuncheon, Republic of Korea

List of supplemental materials

Title	Page
**Supplemental Table S1.** Diagnosis of BPPV	2
**Supplemental Text S1.** Pre-processing and axis measurement for torsional nystagmus	3
**Supplemental Text S2.** Deep learning and grid images for training set	5
**Supplemental video S1**. Diagnostic process using our deep leaning model and eye measurement algorithms	

Supplemental Table S1. Diagnosis of BPPV

Diagnosis	Positional test	Nystagmus patterns
Right posterior canal BPPV	Dix-Hallpike maneuver on right side	Clockwise (CW) torsional- and upbeat-nystagmus after a latency of one or few seconds
Left posterior canal BPPV	Dix-Hallpike maneuver on left side	Counterclockwise (CCW) torsional- and upbeat-nystagmus after a latency of one or few seconds
Right superior canal BPPV	1) Dix-Hallpike on one or both sides<br>2) Lying down or head hanging	Predominantly downbeat- and subtle CW-torsional nystagmus immediately or after a latency of one or few seconds
Left superior canal BPPV	1) Dix-Hallpike on one or both sides<br>2) Lying down or head hanging	Predominantly downbeat- and subtle CCW-torsional nystagmus immediately or after a latency of one or few seconds
Right lateral canal BPPV (geotropic type)	1) Supine roll test<br>2) Bow and lean test	Geotropic nystagmus after a brief latency or no latency<br>Stronger geotropic nystagmus on right supine roll test or right horizontal nystagmus on bow position or left horizontal nystagmus on lean position
Left lateral canal BPPV (geotropic type)	1) Supine roll test<br>2) Bow and lean test	Geotropic nystagmus after a brief latency or no latency<br>Stronger geotropic nystagmus on left supine roll test or left horizontal nystagmus on bow position or right horizontal nystagmus on lean position
Right lateral canal BPPV (ageotropic type)	1) Supine roll test<br>2) Bow and lean test	Ageotropic nystagmus after a brief latency or no latency<br>Weaker ageotropic nystagmus on right supine roll test or left horizontal nystagmus on bow position or right horizontal nystagmus on lean position
Left lateral canal BPPV (ageotropic type)	1) Supine roll test<br>2) Bow and lean test	Ageotropic nystagmus after a brief latency or no latency<br>Weaker ageotropic nystagmus on left supine roll test or right horizontal nystagmus on bow position or left horizontal nystagmus on lean position

Supplemental Text S1. Pre-processing and axis measurement for torsional nystagmus

A template-matching algorithm with a normalized correlation coefficient was applied to calculate differences between patches, which are sliced images of templates with a uniform length, and the reference image. Median or mean differences between the reference and patches were calculated to produce single values representing overall change. The polarity of the torsional movement was determined by summing transient differences over all time points. Except for frames containing median and maximum changes, all frames displaying fast or slow movements were removed to ensure that our algorithm could successfully determine the overall direction of eye movement in each clip.

Transient and overall velocity

A transient velocity of nystagmus was calculated by subtracting the current pupil center with respect to the reference pupil center. The overall velocity of horizontal and vertical nystagmus was determined by a *decision amplitude*, which is the sum of transient velocity values from all the frames. Unlike horizontal, and vertical movements, a transient torsional rotation was measured by tracking changes in the iris striations. An iris area was deemed to be an annulus in Cartesian coordinate; it can be converted to a rectangle in polar coordinate by the following formula:

$$\begin{bmatrix} x \\ y \end{bmatrix} = (\mathrm{R} - \mathrm{r}) \begin{bmatrix} \cos\theta \\ \sin\theta \end{bmatrix}$$

, where R is the outer radius, and r is the inner radius. After the transformation, the rectangle (Fig 1g) was divided into several overlapped patches by which we could calculate vertical difference or distance samples. These samples may contain outlier values at the top and bottom boundaries, which are striation patches around 0 and 2π of the annulus. Aside from them, the template matching algorithm can make mistakes as coincidentally there could be similar patterns coming from the other arcs in the iris. Thus, a median or mean of distance samples was used to reduce the effect of

outliers.

Since in all three-axis measurements, the velocity of pupil and iris area changes non-linearly over time, the differentiation gives the velocity in a certain amount of time.

$$v_m = \frac{\partial P}{\partial t}$$

, where *m* represents the axis of the movement, *P* is the template position of either pupil or iris, and *t* is the time. The changes in velocity of a template over whole time, *T*, can be formulated by the definite integral.

$$\mathrm{S} = \mathrm{s(T)} - \mathrm{s(0)} = \int_0^T v_m$$

The above integral by which the overall displacement of *P* can be approximated by the trapezoidal rule can be noted as follows:

$$\sum_{n=1}^{N} \frac{D(t_{n-1}) + D(t_n)}{2} \Delta t_n$$

, where *N* is the number of frames. *D* denotes the displacement by given time point *t*. The continuous nystagmus is sampled by a uniform time interval, e.g., frames per second. Thus, the above formula can be simplified as follows:

$$\mathrm{S} \approx \frac{D(t_0)}{2} \Delta \mathrm{t} + \sum_{n=1}^{N-1} D(t_n)\, \Delta \mathrm{t} + \frac{D(t_N)}{2} \Delta \mathrm{t}$$

Given that the initial sample serves as the reference position, thus the first term turns to zero. The last term having multiplied by two does not change the overall direction of the nystagmus pattern, meaning that it can be ignored. As the time interval is always same in all the videos, the formula can be far simpler.

$$S \approx \sum_{n=1}^{N} D(t_n)$$

The direction of nystagmus is determined by a fast component, and the velocity below a threshold will be discarded.

Supplemental Text S2. Deep learning and grid images for training set

Among 255^{30} possible combinations on the basis of diagnostic criteria of BPPV, 100,000 grid images were randomly sampled for the training datasets. The 'left' or 'down' direction was represented by values ranging from 0 to 127, and the 'right' or 'up' direction was represented by values ranging from 129 to 255. For instance, the right PSC-BPPV is characterized by upbeating and clockwise torsional nystagmus for the right Dix-Hallpike test. Thus, to generate a grid image representing the right PSC-BPPV, pixel values ranging from 129 to 255 were selected to represent the vertical and torsional nystagmus on the Dix-Hallpike test. All other unrelated grid values were subsequently grayed out. Right ASC-BPPV is characterized by down-beating and right torsional nystagmus when the patient is in the supine head-hanging position or Dix-Hallpike test. Nystagmus is also further enhanced during the left Dix–Hallpike maneuver. Thus, to generate a grid image representing the right ASC-BPPV, pixel values ranging from 0 to 127 and from 129 to 255 were selected, respectively on the positional tests.

Unlike PSC- and ASC-BPPV, the amplitude of direction in samples of LSC-BPPV was lost after data normalization. Hence, 16 amplitudes were re-calculated to recover this information. To reduce the probability of false predictions, nystagmus amplitudes and direction that were unassociated with the relevant BPPV type were grayed out. For example, variables derived from the supine roll test and the bow-and-lean test do not affect the ability of the model to diagnose PSC-BPPV but,

instead, increase the odds of making a false prediction. An L2 regularization with a constant value of 0.001 was applied to reduce overfitting. Batch normalization was performed before rectified linear unit activation. The dropout technique was not used as we did not encounter a significant overfitting issue with L2 regularization while training the model. The softmax function was used to generate the final output layer, producing a prediction with one-hot encoding.

A machine learning algorithm extracts features from a given dataset, $\mathcal{D} = \{x, y\}$, where input data x is given to predict target labels y. There is unsupervised learning by which the features in x are extracted in the way that datasets are given as $\mathcal{D} = \{x, \hat{x}\}$, but, in practice, supervised learning has been prevailing. In supervised learning, the training process evaluates errors based on a loss function $\mathcal{L} = (\mathrm{y}, \hat{\mathrm{y}})$, where $\hat{\mathrm{y}}$ is predicted by the model given input data x. The model parameter, θ, changes as training progresses. The training process is an attempt to find an optimal θ, by which the loss is minimized.

The neural network (NN) is the major structure to build a deep learning model in which neurons are connected through non-linear activation functions, σ, from the input to the output layer.

$$\alpha = \sigma(w^T x + b)$$

Here, w^N denotes the transient weight values of neurons while b represents the bias terms. By both terms, the model parameter is defined as $\theta = \{w, b\}$. The number of hidden layers, N, between the input to the output layer, represents how deep the NN is. The activation of the output layer is dependent on the application, e.g., linear activations for a regression, logistic activations for a binary classification, and softmax activations for a multi-class classification. Recently, the cost or loss optimization has been often done by Adam, adaptive moment estimation [23], optimizer instead of a stochastic gradient descent algorithm. The term stochastic indicates that the training data are split into a set of mini-batches, and randomly shuffled before feeding to the input layer.

In an NN for multi-class classifications, the output layer is mapped to a posterior distribution over classes as follows:

$$\mathrm{P(Y|X;w,b)} = \frac{e^{w^T x+b}}{\sum_k e^{w^T{}_k x+b}}$$

If we assumed a data *x* being generated independently from an identical distribution, the joint probability distribution of data could be obtained by multiplication of individual probabilities, which is equal to the likelihood.

$$\mathcal{L}(\theta|X) = \prod_{k=1} P(y_k|x_k)$$

Applying the logarithm function to the original formula changes multiplications to summations, and reduces exponential terms, leading to simpler calculus of optimization. The maximum likelihood estimation finds the best-fit parameters or weights given *X*, and *Y*. For the negative loglikelihood, the minimization would be applied.

$$\mathcal{O}(\theta) = \mathrm{argmin}\left(-\sum_{k=1} \log(P(y_k|x_k;\theta)\right)$$

A set of weights determined by negative loglikelihood minimization were then stored and used to predict the diagnosis of BPPV.

Softmax activation

Given a dataset X and labels Y, Bayes' theorem can be applied.

$$\mathrm{P(Y|X)} = \frac{P(X|Y)}{P(X)} P(Y)$$

Here, P(Y|X) is posterior probability, $P(X|Y)$ is likelihood or class-conditional density, $P(Y)$ is prior probability, and $P(X)$ is evidence. The prior probability represents what the label distribution

is. The likelihood is the probability distribution of X for each label. The posterior probability is the distribution of Y given data X. After the marginalization, the evidence can be written as follows:

$$\mathrm{P}(X) = \sum_{k} P(X|Y_k)P(Y_k)$$

In a classification problem, the posterior probability is used as a determinant.

$$\mathrm{X}: Y_1 \text{ if } \mathrm{P}(Y_1|\mathrm{X}) > \mathrm{P}(Y_2|\mathrm{X})$$

$$\mathrm{X}: Y_2 \text{ if } \mathrm{P}(Y_1|\mathrm{X}) < \mathrm{P}(Y_2|\mathrm{X})$$

Since the evidence is constant in both rules, the numerator, which is the multiplication of likelihood and prior probability, determines the posterior probability. To simplify the numerator, a logarithm function can be used, which is interchangeable with an exponential function.

$$m_k = \ln(P(X|Y_k)P(Y_k)$$

$$\mathrm{P}(Y_1|\mathrm{X}) = \frac{P(X|Y_1)P(Y_1)}{P(X|Y_1)P(Y_1) + P(X|Y_2)P(Y_2)} = \frac{e^{m_1}}{e^{m_1} + e^{m_2}} = \frac{1}{1 + e^{m_2 - m_1}}$$

The log odds, M, which is a decision boundary value, can be defined.

$$\mathrm{M} = -(m_2 - m_1) = \ln\left(\frac{P(X|Y_1)P(Y_1)}{P(X|Y_2)P(Y_2)}\right)$$

For *k*-class classification problems, the posterior probability of class 1 can be derived from the Bayes' theorem as follows:

,

$$\mathrm{P}(Y_1|\mathrm{X}) = \frac{P(X|Y_1)P(Y_1)}{P(X|Y_1)P(Y_1) + P(X|Y_2)P(Y_2) + \cdots + P(X|Y_k)P(Y_k)} = \frac{e^{m_1}}{\sum_k e^{m_k}}$$

Finally, we can obtain the softmax regression function.

$$\mathrm{P}(\mathrm{Y}|\mathrm{X}) = \frac{e^{\theta_i x}}{\sum_k e^{\theta_k x_k}}$$

, where *i* indicates the class number.
